# *N*′-[(*E*)-4-Benz­yloxy-2-hy­droxy­benzyl­idene]benzohydrazide

**DOI:** 10.1107/S1600536812036306

**Published:** 2012-08-25

**Authors:** P. R. Reshma, M. Sithambaresan, M. R. Prathapachandra Kurup

**Affiliations:** a Department of Applied Chemistry, Cochin University of Science and Technology, Kochi 682 022, India; b Department of Chemistry, Faculty of Science, Eastern University, Sri Lanka, Chenkalady, Sri Lanka

## Abstract

The title compound, C_21_H_18_N_2_O_3_, exists in the *E* conformation with respect to the azomethane C=N double bond. The central benzene ring is almost coplanar with one of the substituent benzene rings [dihedral angle = 1.74 (5)°] and is approximately orthogonal to the other benzene ring of the mol­ecule [dihedral angle = 86.61 (7)°]. An intra­molecular O—H⋯N hydrogen bond occurs. The crystal packing is dominated by N—H⋯O hydrogen bonds, which lead to an infinite chain running parallel to [010].

## Related literature

For the biological activity of hydrazones, see: Patil *et al.* (2010 ▶); Zhang *et al.* (2010 ▶). For the synthesis of related compounds, see: Emmanuel *et al.* (2011 ▶); Mangalam & Kurup (2011 ▶). For related structures, see: Lin & Sang (2009 ▶); Mohd Lair *et al.* (2009 ▶).
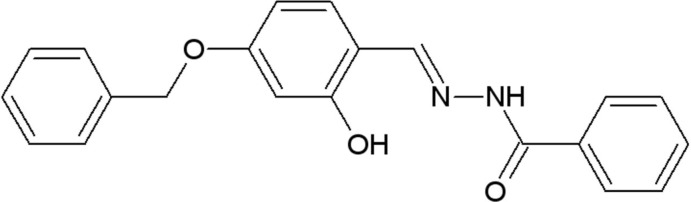


## Experimental

### 

#### Crystal data

C_21_H_18_N_2_O_3_
*M* *_r_* = 346.37
Monoclinic, 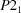

*a* = 10.8053 (6) Å
*b* = 4.8952 (2) Å
*c* = 16.3601 (10) Å
β = 95.813 (2)°
*V* = 860.90 (8) Å^3^
*Z* = 2
Mo *K*α radiation
μ = 0.09 mm^−1^
*T* = 296 K
0.35 × 0.30 × 0.25 mm

#### Data collection

Bruker Kappa APEXII CCD diffractometer
Absorption correction: multi-scan (*SADABS*; Bruker, 2004 ▶) *T*_min_ = 0.969, *T*_max_ = 0.978
9033 measured reflections
1705 independent reflections
1593 reflections with *I* > 2σ(*I*)
*R*_int_ = 0.024

#### Refinement

*R*[*F* ^2^ > 2σ(*F* ^2^)] = 0.029
*wR*(*F* ^2^) = 0.082
*S* = 1.12
1705 reflections
243 parameters
3 restraints
H atoms treated by a mixture of independent and constrained refinement
Δρ_max_ = 0.12 e Å^−3^
Δρ_min_ = −0.14 e Å^−3^

### 

Data collection: *APEX2* (Bruker, 2004 ▶); cell refinement: *APEX2* and *SAINT* (Bruker, 2004 ▶); data reduction: *SAINT* and *XPREP* (Bruker, 2004 ▶); program(s) used to solve structure: *SHELXS97* (Sheldrick, 2008 ▶); program(s) used to refine structure: *SHELXL97* (Sheldrick, 2008 ▶); molecular graphics: *ORTEP-3* (Farrugia, 2012 ▶) and *DIAMOND* (Brandenburg, 2010 ▶); software used to prepare material for publication: *SHELXL97* and *publCIF* (Westrip, 2010 ▶).

## Supplementary Material

Crystal structure: contains datablock(s) I, global. DOI: 10.1107/S1600536812036306/fj2591sup1.cif

Structure factors: contains datablock(s) I. DOI: 10.1107/S1600536812036306/fj2591Isup2.hkl

Supplementary material file. DOI: 10.1107/S1600536812036306/fj2591Isup3.cml

Additional supplementary materials:  crystallographic information; 3D view; checkCIF report

## Figures and Tables

**Table 1:** Hydrogen-bond geometry (Å, °)

*D*—H⋯*A*	*D*—H	H⋯*A*	*D*⋯*A*	*D*—H⋯*A*
N2—H2′⋯O3^i^	0.85 (1)	2.09 (1)	2.903 (2)	160 (2)
O2—H2′′⋯N1	0.87 (2)	1.79 (2)	2.592 (2)	152 (3)

Symmetry code: (i) 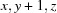.

**Fig. 1.**
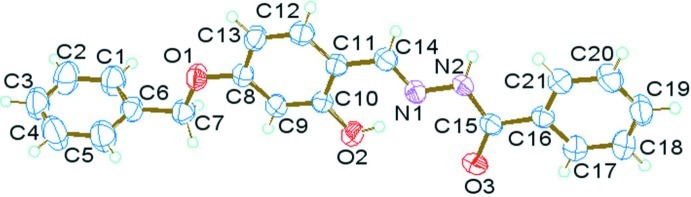
ORTEP view of the unique part of the compound, drawn with 50% probability displacement ellipsoids for the non-H atoms.

**Fig. 2.**
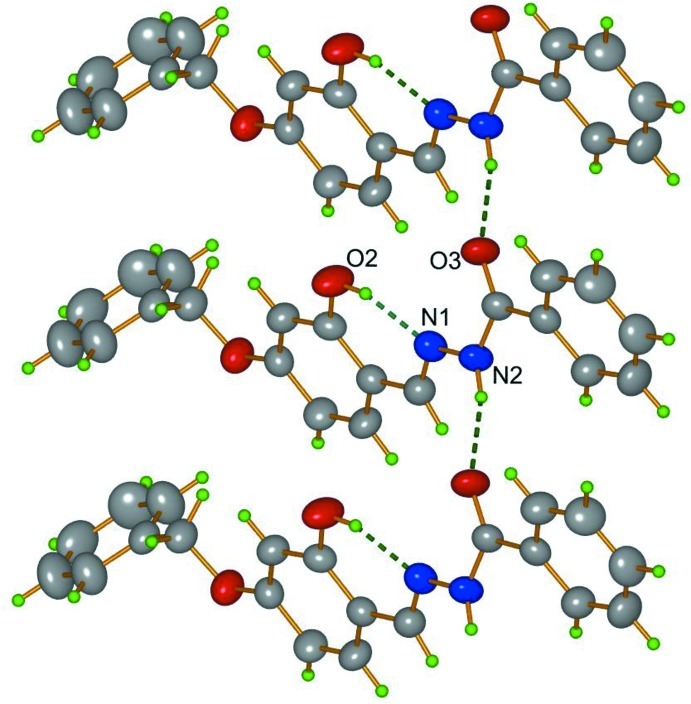
Graphical representation showing one-dimensional supramolecular hydrogen bonding network in the crystal structure of C21H18N2O3.

**Fig. 3.**
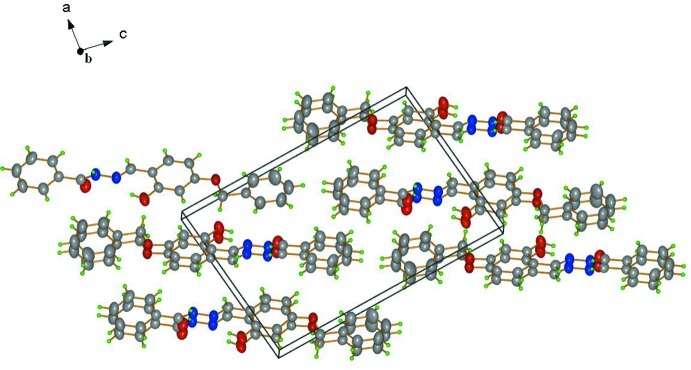
Packing diagram of the compound showing the parallel arrangement of the molecules along b axis.

## supplementary crystallographic information

### Comment

Hydrazone compounds have received much attention due to their potential applications in biological chemistry (Patil *et al.*, 2010; Zhang *et al.*, 2010). As a continuous work on the hydrazone compounds, a new hydrazone compound, *N*'-{(*E*)-[4-(benzyloxy)-2-hydroxyphenyl]methylidene}benzohydrazide, was prepared and structurally characterized. The *ORTEP* view of the title compound is shown in Fig. 1.

The compound crystallizes in monoclinic space group *P*2_1_. The molecule adopts an *E* configuration with respect to C14=N1 bond (Lin & Sang 2009; Mohd Lair *et al.*, 2009) and it exists in amido form with C15=O3 bond length of 1.224 (3) Å which is very close to a formal C=O bond length [1.21 Å]. The aromatic ring C8—C13 is almost coplanar with the ring C16—C21 with dihedral angle of 1.74 (5)° whilst the ring C1—C6 is approximately orthogonal (86.61 (7)°) to the ring C16—C21.

While the intramolecular hydrogen bond O(2)—H(2'')···N(1) increases the rigidity of the molecule, intermolecular N(2)—H(2')···O(3) hydrogen bond (Table 1) links the adjacent molecules forming an infinite one-dimensional supramolecular chain running parallel to the [010] direction in the unit cell (Fig. 2). Benzohydrazone molecules within these chains also interact through very weak π···π interactions with a shortest centroid-centroid distance of 4.8950 (15) Å that not only augment the stronger N—H···O hydrogen bond but also interconnects the infinite chains forming three-dimensional network in the lattice. The parallel arrangement of the molecules along *b* axis is shown in Fig. 3.

### Experimental

The title compound was prepared by adapting a reported procedure (Emmanuel *et al.*, 2011; Mangalam & Kurup, 2011) by refluxing a mixture of methanolic solutions of benzhydrazide (0.136 g,1 mmol) and 4-benzyloxysalicylaldehyde (0.2282 g,1 mmol) for 4 h. The formed crystals were collected, washed with few drops of methanol and dried over P_4_O_10_*in vacuo*. Single crystals of the title compound suitable for X-ray analysis were obtained by slow evaporation from its methanolic solution.

### Refinement

All H atoms on C were placed in calculated positions, guided by difference maps, with C—H bond distances 0.93–0.97 Å. H atoms were assigned as *U*_iso_=1.2Ueq. H atoms of O2—H2'' and N2—H2' bonds were located from difference maps and restrained using *DFIX* instructions with O—H = 0.87 ± 0.02 Å and N—H = 0.85 ± 0.01 Å.

In the absence of significant anomalous scattering effects Friedel pairs have been merged.

### Crystal data

**Table:**

C_21_H_18_N_2_O_3_	*F*(000) = 364
*M**_r_* = 346.37	*D*_x_ = 1.336 Mg m^−^^3^
Monoclinic, *P*2_1_	Mo *K*α radiation, λ = 0.71073 Å
Hall symbol: P 2yb	Cell parameters from 5951 reflections
*a* = 10.8053 (6) Å	θ = 2.4–28.1°
*b* = 4.8952 (2) Å	µ = 0.09 mm^−^^1^
*c* = 16.3601 (10) Å	*T* = 296 K
β = 95.813 (2)°	Block, colorless
*V* = 860.90 (8) Å^3^	0.35 × 0.30 × 0.25 mm
*Z* = 2	

### Data collection

**Table:**

Bruker Kappa APEXII CCD diffractometer	1705 independent reflections
Radiation source: fine-focus sealed tube	1593 reflections with *I* > 2σ(*I*)
Graphite monochromator	*R*_int_ = 0.024
Detector resolution: 8.33 pixels mm^-1^	θ_max_ = 25.0°, θ_min_ = 3.0°
ω and φ scan	*h* = −12→12
Absorption correction: multi-scan (*SADABS*; Bruker, 2004)	*k* = −5→5
*T*_min_ = 0.969, *T*_max_ = 0.978	*l* = −19→19
9033 measured reflections	

### Refinement

**Table:**

Refinement on *F*^2^	Primary atom site location: structure-invariant direct methods
Least-squares matrix: full	Secondary atom site location: difference Fourier map
*R*[*F*^2^ > 2σ(*F*^2^)] = 0.029	Hydrogen site location: inferred from neighbouring sites
*wR*(*F*^2^) = 0.082	H atoms treated by a mixture of independent and constrained refinement
*S* = 1.12	*w* = 1/[σ^2^(*F*_o_^2^) + (0.0492*P*)^2^ + 0.0573*P*] where *P* = (*F*_o_^2^ + 2*F*_c_^2^)/3
1705 reflections	(Δ/σ)_max_ = 0.005
243 parameters	Δρ_max_ = 0.12 e Å^−^^3^
3 restraints	Δρ_min_ = −0.14 e Å^−^^3^

### Special details

**Table:**

Geometry. All e.s.d.'s (except the e.s.d. in the dihedral angle between two l.s. planes) are estimated using the full covariance matrix. The cell e.s.d.'s are taken into account individually in the estimation of e.s.d.'s in distances, angles and torsion angles; correlations between e.s.d.'s in cell parameters are only used when they are defined by crystal symmetry. An approximate (isotropic) treatment of cell e.s.d.'s is used for estimating e.s.d.'s involving l.s. planes.
Refinement. Refinement of *F*^2^ against ALL reflections. The weighted *R*-factor *wR* and goodness of fit *S* are based on *F*^2^, conventional *R*-factors *R* are based on *F*, with *F* set to zero for negative *F*^2^. The threshold expression of *F*^2^ > σ(*F*^2^) is used only for calculating *R*-factors(gt) *etc*. and is not relevant to the choice of reflections for refinement. *R*-factors based on *F*^2^ are statistically about twice as large as those based on *F*, and *R*- factors based on ALL data will be even larger.

### Fractional atomic coordinates and isotropic or equivalent isotropic displacement parameters (Å^2^)

**Table:**

	*x*	*y*	*z*	*U*_iso_*/*U*_eq_	
O1	0.08187 (12)	0.6299 (3)	1.18699 (7)	0.0525 (4)	
O2	0.19313 (15)	0.4504 (4)	0.91841 (8)	0.0610 (4)	
O3	0.43474 (14)	0.3828 (3)	0.76116 (9)	0.0575 (4)	
N1	0.37436 (15)	0.7368 (4)	0.87075 (9)	0.0471 (4)	
N2	0.44743 (17)	0.8127 (3)	0.81016 (10)	0.0458 (4)	
C1	−0.1620 (2)	0.5457 (6)	1.27899 (13)	0.0681 (7)	
H1	−0.1984	0.6716	1.2413	0.082*	
C2	−0.2072 (2)	0.5169 (8)	1.35467 (15)	0.0812 (9)	
H2A	−0.2737	0.6235	1.3677	0.097*	
C3	−0.1550 (3)	0.3347 (7)	1.40967 (15)	0.0779 (8)	
H3	−0.1853	0.3174	1.4607	0.093*	
C4	−0.0583 (3)	0.1761 (8)	1.39081 (16)	0.0890 (9)	
H4	−0.0228	0.0501	1.4288	0.107*	
C5	−0.0126 (2)	0.2025 (7)	1.31467 (15)	0.0750 (7)	
H5	0.0528	0.0928	1.3015	0.090*	
C6	−0.06386 (17)	0.3899 (5)	1.25910 (11)	0.0500 (5)	
C7	−0.01383 (18)	0.4243 (5)	1.17735 (11)	0.0531 (5)	
H7A	0.0203	0.2531	1.1600	0.064*	
H7B	−0.0798	0.4808	1.1361	0.064*	
C8	0.14629 (15)	0.6828 (4)	1.12181 (10)	0.0424 (4)	
C9	0.13243 (17)	0.5416 (4)	1.04847 (11)	0.0464 (5)	
H9	0.0725	0.4054	1.0401	0.056*	
C10	0.20779 (17)	0.6026 (4)	0.98724 (10)	0.0431 (4)	
C11	0.29517 (17)	0.8163 (4)	0.99739 (10)	0.0421 (4)	
C12	0.30438 (17)	0.9593 (5)	1.07174 (11)	0.0501 (5)	
H12	0.3604	1.1034	1.0794	0.060*	
C13	0.23345 (17)	0.8937 (5)	1.13355 (11)	0.0493 (5)	
H13	0.2431	0.9886	1.1830	0.059*	
C14	0.37200 (17)	0.8897 (5)	0.93334 (11)	0.0470 (4)	
H14	0.4195	1.0484	0.9380	0.056*	
C15	0.47098 (17)	0.6182 (4)	0.75527 (11)	0.0427 (4)	
C16	0.54733 (16)	0.7030 (4)	0.68911 (11)	0.0433 (4)	
C17	0.5333 (2)	0.5618 (5)	0.61561 (12)	0.0565 (6)	
H17	0.4738	0.4244	0.6077	0.068*	
C18	0.6065 (2)	0.6223 (6)	0.55402 (13)	0.0671 (6)	
H18	0.5957	0.5273	0.5046	0.080*	
C19	0.6950 (2)	0.8216 (6)	0.56521 (14)	0.0673 (7)	
H19	0.7449	0.8610	0.5236	0.081*	
C20	0.7105 (2)	0.9643 (6)	0.63802 (14)	0.0655 (6)	
H20	0.7709	1.0998	0.6455	0.079*	
C21	0.63653 (18)	0.9069 (5)	0.70001 (13)	0.0532 (5)	
H21	0.6466	1.0048	0.7489	0.064*	
H2'	0.4593 (19)	0.9831 (9)	0.8048 (13)	0.047 (6)*	
H2''	0.249 (2)	0.514 (7)	0.8883 (16)	0.100 (10)*	

### Atomic displacement parameters (Å^2^)

**Table:**

	*U*^11^	*U*^22^	*U*^33^	*U*^12^	*U*^13^	*U*^23^
O1	0.0541 (7)	0.0627 (10)	0.0425 (6)	−0.0105 (7)	0.0144 (5)	−0.0043 (7)
O2	0.0859 (10)	0.0528 (10)	0.0474 (7)	−0.0177 (8)	0.0207 (7)	−0.0110 (7)
O3	0.0747 (9)	0.0345 (8)	0.0668 (9)	−0.0058 (7)	0.0238 (7)	0.0026 (7)
N1	0.0560 (9)	0.0423 (10)	0.0454 (8)	0.0030 (8)	0.0161 (7)	0.0061 (8)
N2	0.0602 (9)	0.0320 (9)	0.0478 (8)	0.0002 (7)	0.0184 (7)	0.0059 (7)
C1	0.0652 (12)	0.0807 (18)	0.0605 (12)	0.0120 (13)	0.0162 (10)	0.0076 (13)
C2	0.0728 (15)	0.103 (2)	0.0724 (15)	0.0086 (16)	0.0311 (12)	−0.0023 (17)
C3	0.0884 (17)	0.090 (2)	0.0604 (13)	−0.0181 (17)	0.0300 (12)	0.0016 (15)
C4	0.106 (2)	0.094 (2)	0.0688 (15)	0.0056 (19)	0.0186 (14)	0.0313 (17)
C5	0.0768 (15)	0.0803 (19)	0.0712 (14)	0.0118 (14)	0.0235 (12)	0.0150 (14)
C6	0.0466 (10)	0.0556 (12)	0.0488 (9)	−0.0096 (10)	0.0094 (8)	−0.0032 (10)
C7	0.0530 (10)	0.0581 (14)	0.0497 (10)	−0.0071 (11)	0.0115 (8)	−0.0053 (10)
C8	0.0418 (9)	0.0464 (12)	0.0396 (9)	0.0028 (8)	0.0060 (7)	0.0006 (9)
C9	0.0505 (10)	0.0439 (11)	0.0454 (10)	−0.0058 (9)	0.0078 (8)	0.0003 (9)
C10	0.0536 (10)	0.0382 (10)	0.0377 (8)	0.0020 (9)	0.0059 (7)	0.0002 (8)
C11	0.0447 (9)	0.0407 (11)	0.0414 (9)	0.0028 (8)	0.0061 (7)	0.0016 (8)
C12	0.0504 (10)	0.0492 (13)	0.0508 (10)	−0.0105 (10)	0.0055 (8)	−0.0052 (9)
C13	0.0542 (10)	0.0547 (12)	0.0391 (9)	−0.0051 (10)	0.0055 (7)	−0.0061 (10)
C14	0.0487 (9)	0.0425 (11)	0.0507 (10)	−0.0011 (9)	0.0091 (8)	0.0022 (10)
C15	0.0482 (10)	0.0349 (10)	0.0460 (9)	0.0024 (9)	0.0089 (7)	0.0057 (9)
C16	0.0482 (10)	0.0364 (10)	0.0465 (9)	0.0053 (8)	0.0097 (8)	0.0052 (8)
C17	0.0673 (12)	0.0523 (14)	0.0515 (11)	−0.0063 (11)	0.0139 (9)	−0.0026 (10)
C18	0.0844 (15)	0.0710 (17)	0.0487 (11)	−0.0028 (15)	0.0210 (10)	−0.0012 (12)
C19	0.0731 (14)	0.0721 (17)	0.0613 (13)	0.0023 (13)	0.0296 (11)	0.0135 (13)
C20	0.0601 (12)	0.0620 (16)	0.0779 (15)	−0.0129 (12)	0.0245 (10)	0.0058 (13)
C21	0.0565 (10)	0.0488 (12)	0.0558 (10)	−0.0040 (10)	0.0131 (8)	0.0003 (10)

### Geometric parameters (Å, º)

**Table:**

O1—C8	1.356 (2)	C8—C9	1.380 (3)
O1—C7	1.440 (3)	C8—C13	1.397 (3)
O2—C10	1.346 (2)	C9—C10	1.386 (3)
O2—H2''	0.871 (18)	C9—H9	0.9300
O3—C15	1.224 (3)	C10—C11	1.407 (3)
N1—C14	1.271 (2)	C11—C12	1.398 (3)
N1—N2	1.379 (2)	C11—C14	1.447 (2)
N2—C15	1.350 (3)	C12—C13	1.367 (3)
N2—H2'	0.8500 (11)	C12—H12	0.9300
C1—C6	1.372 (3)	C13—H13	0.9300
C1—C2	1.383 (3)	C14—H14	0.9300
C1—H1	0.9300	C15—C16	1.485 (2)
C2—C3	1.349 (4)	C16—C17	1.382 (3)
C2—H2A	0.9300	C16—C21	1.386 (3)
C3—C4	1.363 (4)	C17—C18	1.375 (3)
C3—H3	0.9300	C17—H17	0.9300
C4—C5	1.392 (3)	C18—C19	1.365 (4)
C4—H4	0.9300	C18—H18	0.9300
C5—C6	1.369 (4)	C19—C20	1.376 (3)
C5—H5	0.9300	C19—H19	0.9300
C6—C7	1.502 (2)	C20—C21	1.382 (3)
C7—H7A	0.9700	C20—H20	0.9300
C7—H7B	0.9700	C21—H21	0.9300
			
C8—O1—C7	117.87 (14)	O2—C10—C9	117.22 (18)
C10—O2—H2''	104 (2)	O2—C10—C11	122.05 (16)
C14—N1—N2	118.70 (17)	C9—C10—C11	120.73 (17)
C15—N2—N1	116.64 (16)	C12—C11—C10	117.55 (16)
C15—N2—H2'	125.7 (15)	C12—C11—C14	120.68 (18)
N1—N2—H2'	116.2 (15)	C10—C11—C14	121.77 (17)
C6—C1—C2	120.5 (2)	C13—C12—C11	121.99 (19)
C6—C1—H1	119.8	C13—C12—H12	119.0
C2—C1—H1	119.8	C11—C12—H12	119.0
C3—C2—C1	120.2 (3)	C12—C13—C8	119.49 (17)
C3—C2—H2A	119.9	C12—C13—H13	120.3
C1—C2—H2A	119.9	C8—C13—H13	120.3
C2—C3—C4	120.3 (2)	N1—C14—C11	119.8 (2)
C2—C3—H3	119.9	N1—C14—H14	120.1
C4—C3—H3	119.9	C11—C14—H14	120.1
C3—C4—C5	120.0 (3)	O3—C15—N2	121.87 (17)
C3—C4—H4	120.0	O3—C15—C16	121.75 (18)
C5—C4—H4	120.0	N2—C15—C16	116.34 (18)
C6—C5—C4	120.0 (3)	C17—C16—C21	119.04 (17)
C6—C5—H5	120.0	C17—C16—C15	118.32 (18)
C4—C5—H5	120.0	C21—C16—C15	122.54 (18)
C5—C6—C1	119.1 (2)	C18—C17—C16	120.6 (2)
C5—C6—C7	120.6 (2)	C18—C17—H17	119.7
C1—C6—C7	120.4 (2)	C16—C17—H17	119.7
O1—C7—C6	107.46 (16)	C19—C18—C17	120.2 (2)
O1—C7—H7A	110.2	C19—C18—H18	119.9
C6—C7—H7A	110.2	C17—C18—H18	119.9
O1—C7—H7B	110.2	C18—C19—C20	120.06 (19)
C6—C7—H7B	110.2	C18—C19—H19	120.0
H7A—C7—H7B	108.5	C20—C19—H19	120.0
O1—C8—C9	124.68 (17)	C19—C20—C21	120.2 (2)
O1—C8—C13	115.18 (16)	C19—C20—H20	119.9
C9—C8—C13	120.13 (16)	C21—C20—H20	119.9
C8—C9—C10	120.04 (18)	C20—C21—C16	119.9 (2)
C8—C9—H9	120.0	C20—C21—H21	120.1
C10—C9—H9	120.0	C16—C21—H21	120.1
			
C14—N1—N2—C15	164.71 (18)	C10—C11—C12—C13	1.2 (3)
C6—C1—C2—C3	−0.1 (4)	C14—C11—C12—C13	−179.66 (19)
C1—C2—C3—C4	−0.6 (5)	C11—C12—C13—C8	−1.9 (3)
C2—C3—C4—C5	0.3 (5)	O1—C8—C13—C12	178.99 (18)
C3—C4—C5—C6	0.7 (5)	C9—C8—C13—C12	0.2 (3)
C4—C5—C6—C1	−1.4 (4)	N2—N1—C14—C11	179.39 (16)
C4—C5—C6—C7	178.8 (3)	C12—C11—C14—N1	170.89 (18)
C2—C1—C6—C5	1.0 (4)	C10—C11—C14—N1	−10.0 (3)
C2—C1—C6—C7	−179.1 (3)	N1—N2—C15—O3	−3.6 (3)
C8—O1—C7—C6	175.83 (17)	N1—N2—C15—C16	178.67 (15)
C5—C6—C7—O1	−90.4 (3)	O3—C15—C16—C17	28.2 (3)
C1—C6—C7—O1	89.8 (2)	N2—C15—C16—C17	−154.05 (19)
C7—O1—C8—C9	−4.4 (3)	O3—C15—C16—C21	−148.1 (2)
C7—O1—C8—C13	176.89 (17)	N2—C15—C16—C21	29.7 (3)
O1—C8—C9—C10	−176.51 (17)	C21—C16—C17—C18	−0.1 (3)
C13—C8—C9—C10	2.1 (3)	C15—C16—C17—C18	−176.5 (2)
C8—C9—C10—O2	177.42 (18)	C16—C17—C18—C19	0.7 (4)
C8—C9—C10—C11	−2.9 (3)	C17—C18—C19—C20	−0.6 (4)
O2—C10—C11—C12	−179.1 (2)	C18—C19—C20—C21	−0.1 (4)
C9—C10—C11—C12	1.2 (3)	C19—C20—C21—C16	0.6 (4)
O2—C10—C11—C14	1.8 (3)	C17—C16—C21—C20	−0.5 (3)
C9—C10—C11—C14	−177.93 (18)	C15—C16—C21—C20	175.6 (2)

### Hydrogen-bond geometry (Å, º)

**Table:**

*D*—H···*A*	*D*—H	H···*A*	*D*···*A*	*D*—H···*A*
N2—H2′···O3^i^	0.85 (1)	2.09 (1)	2.903 (2)	160 (2)
O2—H2′′···N1	0.87 (2)	1.79 (2)	2.592 (2)	152 (3)

Symmetry code: (i) *x*, *y*+1, *z*.

## References

[bb1] Brandenburg, K. (2010). *DIAMOND* Crystal Impact GbR, Bonn, Germany.

[bb2] Bruker (2004). *SADABS*, *APEX2*, *XPREP* and *SAINT* Bruker AXS Inc., Madison, Wisconsin, USA.

[bb3] Emmanuel, J., Sithambaresan, M. & Kurup, M. R. P. (2011). *Acta Cryst.* E**67**, o3267. 10.1107/S1600536811046381 PMC3238925 22199774

[bb4] Farrugia, L. J. (2012). *J. Appl. Cryst.* **45**, 849–854.

[bb5] Lin, X.-S. & Sang, Y.-L. (2009). *Acta Cryst.* E**65**, o1650. 10.1107/S1600536809022983 PMC2969367 21582914

[bb6] Mangalam, N. A. & Kurup, M. R. P. (2011). *Spectrochim. Acta Part A*, **76**, 22–28.

[bb7] Mohd Lair, N., Mohd Ali, H. & Ng, S. W. (2009). *Acta Cryst.* E**65**, o190. 10.1107/S160053680804289X PMC2968098 21581645

[bb8] Patil, S. A., Naik, V. H., Kulkarni, A. D., Kamble, U., Bagihalli, G. B. & Badami, P. S. (2010). *J. Coord. Chem.* **63**, 688–699.

[bb9] Sheldrick, G. M. (2008). *Acta Cryst.* A**64**, 112–122. 10.1107/S0108767307043930 18156677

[bb10] Westrip, S. P. (2010). *J. Appl. Cryst.* **43**, 920–925.

[bb11] Zhang, Y. H., Zhang, L., Liu, L., Guo, J. X., Wu, D. L., Xu, G. C., Wang, X. H. & Jia, D. Z. (2010). *Inorg. Chim. Acta*, **363**, 289–293.

